# Perioperative safety of two-team simultaneous bilateral total knee arthroplasty in the obese patient

**DOI:** 10.1186/1749-799X-5-38

**Published:** 2010-06-17

**Authors:** Benjamin C Taylor, Craig Dimitris, John G Mowbray, Steven T Gaines, Robert N Steensen

**Affiliations:** 1 Department of Orthopaedic Surgery, Mount Carmel Medical Center, MSB 3rd Floor, 793 W. State Street, Columbus, Ohio, 43222, USA; 2 Cardinal Orthopaedic Institute, 3777 Trueman Court, Hilliard, OH, 43026, USA

## Abstract

**Background:**

Although the rates of perioperative morbidity and mortality with simultaneous bilateral total knee arthroplasty remain a concern, multiple studies have shown the procedure to be safe in selected patient populations. Evidence also remains mixed regarding the outcomes of total knee arthroplasty in obese patients. The purpose of this paper is to compare the rates of perioperative morbidity and mortality in consecutive obese patients undergoing two-team simultaneous bilateral total knee arthroplasty and unilateral total knee arthroplasty.

**Methods:**

The records on all two-team simultaneous total knee arthroplasties and unilateral total knee arthroplasties from October 1997 to December 2007 were reviewed. A total of 151 patients with a body mass index (BMI) >30 undergoing two-team simultaneous total knee arthroplasty and 148 patients with a BMI >30 undergoing unilateral total knee arthroplasty were retrospectively reviewed and analyzed to determine perioperative morbidity and mortality as well as one-year mortality rates.

**Results:**

Preoperative patient characteristics did not show any significant differences between groups. The simultaneous bilateral group had significantly longer operative times (127.4 versus 112.7 minutes, p < 0.01), estimated blood loss (176.7 versus 111.6 mL, p = 0.01), percentage of patients requiring blood transfusion (64.9% versus 13.9%, p < 0.01), length of hospital stay (3.72 versus 3.30 days, p < 0.01), and percentage of patients requiring extended care facility usage at discharge (63.6% versus 27.8%, p < 0.01). No significant difference between unilateral and bilateral groups was seen in regards to total complication rate, major or minor complication subgroup rate, or any particular complication noted. Doubling the variables in the unilateral group for a staged total knee arthroplasty scenario did create significant increases over the simultaneous data in almost every data category.

**Conclusions:**

Two-team simultaneous total knee arthroplasty appears to be safe in obese patients, with similar complication rates as compared to unilateral procedures. Two-team simultaneous total knee arthroplasty also appears to have potential benefits over a staged procedure in the obese patient, although more study is required regarding this topic.

## Background

Total knee arthroplasty (TKA) is a successful, reproducible procedure in patients with osteoarthritis [1]. The prevalence of bilateral knee osteoarthritis has been shown to be as high as 5%, which forces the patient and physician to confront the dilemma of whether to undergo TKA as a 1- or 2- stage procedure [2].

There are 3 options for the timing of surgery: staged, sequential, or simultaneous. However, the orthopaedic literature has been inconsistent in defining this important terminology. The authors have attempted to clarify these terms using the following definitions. A staged procedure involves 2 unilateral arthroplasties, performed during separate anesthesias, frequently over 2 separate inpatient stays. In contrast, sequential arthroplasties are performed by 1 surgical team with the patient under 1 anesthetic. Truly simultaneous bilateral procedures are performed concurrently by 2 surgical teams with the patient under 1 anesthetic.

The decision to proceed with simultaneous bilateral TKA carries both risks and benefits beyond those of staged arthroplasty. There are reports of increased perioperative morbidity, including pulmonary, cardiac, neurologic, gastrointestinal and wound complications, deep vein thrombosis, pulmonary embolism, and intensive care unit admissions [3-11]. Although this information is valuable, these published studies unfortunately describe a variety of staged, sequential, and 2-team simultaneous approaches. Bilateral TKAs performed during the same inpatient stay result in higher patient satisfaction, shorter overall rehabilitation time, and decreased cost [12]. However, despite the proliferation of published work relating to this topic, no absolute statement can be made regarding the relative risk of undergoing simultaneous bilateral TKA because of inconsistent terminology and variable study design.

Obesity (body mass index ≥30 kg/m^2^) is a strong predictor of bilateral osteoarthritis of the knee [2]. With estimates of some populations worldwide having obesity rates near 50% and at least 300 million people worldwide thought to be obese, this clinical situation arises with increased frequency [13,14]. The effects of obesity on patient outcomes after unilateral TKA have varied substantially, revealing conflicting data regarding patient-centered outcome surveys, infection rate, revision rate, perioperative morbidity and short-term mortality [15-22].

The purpose of our retrospective study was to compare 2-team simultaneous and unilateral TKA in the obese population in terms of perioperative complications. We hypothesized that there would be no significant differences in perioperative morbidity or mortality in the first year post TKA.

## Methods

### Patient Population

After institutional review board approval was obtained, the surgical records of a single tertiary hospital were reviewed to retrospectively identify all patients who underwent unilateral or simultaneous bilateral TKA by the senior authors (STG and RNS) between October 1997 and December 2007. Patients were excluded if their preoperative body mass index (BMI) was <30 kg/m^2^, if an additional concurrent procedure was performed, if the procedure was a revision arthroplasty, or if a 2-stage procedure for septic arthritis was performed. After review of the surgical records, 151 simultaneous bilateral TKAs and 148 unilateral TKAs met the inclusion criteria.

### Perioperative Procedures

The simultaneous arthroplasties were performed by 2 surgical teams with the patient under a single anesthetic. Each team consisted of an attending surgeon, a surgical technician, and an orthopaedic surgical resident, medical student, or surgical assistant. The 2 senior authors (STG and RNS) were the primary surgeons in each procedure. Tourniquets were utilized in all cases, with inflation times staggered by 5 minutes and deflation times staggered by at least 5 minutes side by side. An intramedullary guide was used for femoral alignment, and an intramedullary and/or extramedullary guide was utilized for tibial alignment at the surgeon's discretion. Each patella was evaluated for possible resurfacing. Each posterior cruciate ligament was evaluated, and the appropriate posterior-stabilized or standard cruciate-retaining prostheses were selected (Biomet, Warsaw, IN; Smith & Nephew, Memphis, TN; and DePuy, Warsaw, IN). All components were cemented with methylmethacrylate (DePuy, Warsaw, IN). Blood loss was estimated by both primary surgeons and anesthesia staff using clinical judgement as well as analysis of blood in the suction canisters, surgical towels, and the remainder of the surgical field; a final estimated blood loss was then listed after consensus was obtained between the three parties.

Standardized postoperative clinical pathways were utilized throughout the time period of this study. Continuous passive motion was initiated in the post-anesthesia care unit and used throughout the patient's hospital stay. Cefazolin (clindamycin if penicillin-allergic) antibiotic coverage was extended for 24 hours postoperatively. Oral as well as parental narcotics were utilized for pain control in the majority of patients. Dressings were changed on postoperative day 2 and each day thereafter. On postoperative day 1, physical therapy was initiated, as was an assessment of discharge planning needs. Anticoagulation consisted of either warfarin, enoxaparin, or combination enoxaparin and warfarin therapy. Most patients were discharged to home or to a rehabilitation facility on the 3^rd ^or 4^th ^postoperative day. Indication of manipulation for the arthrofibrotic knees in this patient group was active flexion of less than 80° to 90°, after other treatment options were maximized, including an aggressive physical therapy program with adequate pain control

### Chart Review

Hospital as well as office records were reviewed retrospectively in order to acquire the perioperative data under investigation. Surgical parameters were recorded from the anesthesiology record, operative report, and surgical nurse's notes. The remainder of the hospital stay was reviewed via the electronic hospital record; physician records were also reviewed for follow-up through the 1^st ^year to ensure collection of appropriate data. All perioperative complications were recorded and classified as either minor or major complications. Minor complications included urinary retention or infection, superficial infections, or deep venous thrombosis diagnosed by Doppler ultrasonography. Major complications included deep infections, pulmonary embolism, cerebrovascular accident, myocardial infarction, death, or a return to the operating room for any reason.

### Statistical Methods

Continuous variables were analyzed for significance using the Student t test with Microsoft Excel software (Redmond, WA). A Fisher exact test or chi-square analysis was used for analysis of dichotomous variables. All confidence intervals were calculated at the level of 95% and significance was determined as *P *< .05.

## Results

During the 10-year study period, a total of 151 obese patients underwent unilateral TKA and 148 obese patients underwent simultaneous, 2-team TKA. Clinical characteristics between the 2 groups did not differ significantly, with the exception of a greater percentage of men in the unilateral group (Table 1).

**Table 1:** Patient and Operative Data

Characteristic	Unilateral	Bilateral	*P *value
Number of patients (knees)	151	148	N/A
Age in years	63.8 ± 8.4 (44-84)	65.1 ± 9.1 (37-87)	.209
Sex (M:F)(M%)	52:99 (34.4%)	41:107 (27.7%)	.045
BMI (kg/m^2^)	37. 1 ± 6.6 (30.0-67.8)	37. 7 ± 5.9 (30.2-61.4)	.41
ASA Classification^a ^(mean)	2.9 ± 0.5	2.8 ± 0.6	0.13
1 (%)	0 (0%)	1 (0.7%)	-
2 (%)	30 (20.9%)	41 (27.2%)	.11
3 (%)	100 (70.3%)	99 (65.6%)	.90
4 (%)	13 (8.8%)	10 (6.7%)	.55
Operative time (min)	112.7 ± 20.6 (77-181)	127.4 ± 19.7 (92-223)	< 0.01
Tourniquet time (min)	110.4 ± 18.0 (71-155)	116.0 ± 18.8 (17-176)	.01
Estimated blood loss (mL)	111.6 ± 117.3 (10-1000)	176.7 ± 249.7 (25-2500)	.01
Change in hemoglobin at discharge (gm/dL)	-3.1 ± 1.1 (-5.7 - 0)	-2.9 ± 1.6 (-7.6 - +2)	.23
Patients transfused	21 (13.9%)	96 (64.9%)	< .01
Units transfused (mean)	0.3 ± 0.7 (0 - 4)	1.5 ± 1.4 (0 - 6)	< .01
Length of hospital stay (days)	3.30 ± 0.72 (2 - 7)	3.72 ± 1.09 (2 - 7)	< .01
To extended care facility at discharge (percentage)	42 (27.8%)	96 (63.6%)	< .01
Mean duration of follow-up (months)	14.9 ± 10.5 (4 - 55)	15.1 ± 10.5 (3 - 48)	.90

^a ^ASA Classification: American Society of Anesthesiologists physical status classification system.

Operative variables did have some significant differences, including significant increases in operative time for bilateral cases (127.4 minutes vs. 112.7 minutes, *P *< .01), tourniquet time (116.0 minutes vs. 110.4 minutes, *P *= .01), and estimated blood loss (176.7 mL vs. 111.6 mL, *P *= .01). Intraoperative crystalloid replacement was significantly greater in the bilateral group (2293.96 mL vs. 2059.38 mL, *P *< .01). Postoperatively, 21 unilateral patients (13.9%) and 96 bilateral patients (64.9%) required transfusion (*P *< .01). Blood transfusion was given to those patients who became cardiovascularly unstable or whose hemoglobin level fell below 8 g/dL postoperatively. Although there was a statistically significant increase in the proportion of patients receiving perioperative transfusions (*P *< .01) and a higher number of mean units transfused (*P *< .01) in the bilateral group, there was no significant difference in change between preoperative and postoperative hemoglobin levels between the two groups (*P *= .23). Mean hospital stay was significantly longer for the bilateral group as compared to the unilateral group (3.72 days vs. 3.30 days, respectively, (*P *< .01). Similarly, significantly more bilateral patients were discharged to an extended-care facility (n = 96, 63.6% vs. n = 41, 27.8%, *P *< .01). Additional operative data are shown in Table 1.

Major complications occurred in 8.6% (n = 13) of the bilateral patient group and 5.4% (n = 8) of the unilateral group, a nonsignificant difference (*P *= .28) (Table 2). There were no significant differences between groups for the occurrence of major complications (i.e., death within 6 months, pulmonary embolism, myocardial infarction, congestive heart failure, cerebrovascular accident, acute renal insufficiency, need for implant revision, and/or the need for further operative treatment of the knee). Reasons for further surgery in the bilateral group included two intraarticular snapping popliteal tendons and one traumatic patellar dislocation; reasons for further surgery in the unilateral group included wound necrosis requiring a flap, superficial abscess needing superficial debridement, one snapping popliteal tendon, and extensor mechanism rupture. There was a slight, yet nonsignificant trend for more minor complications (i.e., superficial infection, distal deep venous thrombosis, urinary tract infection, urinary retention, confusion, ileus, surgical hematoma, and need for knee manipulation) in the unilateral patient group (n = 30, 20.3% vs. n = 19, 12.6%, *P *= 0.07). The mean follow-up was 14.9 months for the unilateral group and 15.1 months for the bilateral group. Functionally, the groups were also very similar in terms of final knee range of motion (ROM) at the final follow-up evaluation, with unilateral and bilateral patients having a range of motion of 1° - 116° and 0° - 114°, respectively.

**Table 2:** Major and minor complications

Complications	Bilateral (n = 151) Number (%)	Unilateral (n = 148) Number (%)	*P*
**Major**	13 (8.6%)	8 (5.4%)	.28
Death	0 (0%)	0 (0%)	-
Pulmonary embolism	4 (2.6%)	1 (0.7%)	.56
Myocardial infarction	1 (0.7%)	0	.32
Deep infection	1 (0.7%)	1 (0.7%)	.96
Congestive heart failure	0	1 (0.7%)	.50
Cerebrovascular accident	0	0	-
Acute renal insufficiency	3 (2.0%)	0	.25
Revision of implant	1 (0.7%)	1 (0.7%)	.96
Any need for further surgery on involved knee (not including manipulation)	3 (2.0%)	4 (2.7%)	.68
**Minor**	19 (12.6%)	30 (20.3%)	.07
Superficial infection	1 (0.7%)	4 (2.7%)	.17
Distal deep-vein thrombosis	3 (2.0%)	3 (2.0%)	.98
Urinary tract infection	2 (1.3%)	2 (1.4%)	.98
Urinary retention	3 (2.0%)	4 (2.7%)	.68
Confusion	2 (1.3%)	2 (1.4%)	.98
Ileus	2 (1.3%)	4 (2.7%)	.40
Surgical hematoma	2 (1.3%)	2 (1.4%)	.98
Need for knee manipulation	4 (2.6%)	9 (6.1%)	.15

## Discussion

Controversy remains regarding the relative safety of simultaneous bilateral TKA performed with the patient under 1 anesthetic [3-12]. Unfortunately, published studies describe a variety of staged, sequential, and 2-team simultaneous approaches, which prevents valid comparison between studies. To complicate the topic further, many conflicting reports exist concerning the effect of obesity on the risk of short- and long-term complications in patients undergoing unilateral primary TKA [15-22]. We have attempted to carefully assess outcomes in obese patients undergoing simultaneous, 2-team bilateral TKA and compare them with a matched cohort of obese patients undergoing unilateral TKA.

We believe that we are the first study comparing these 2 cohorts undergoing 2-team, simultaneous bilateral TKA and unilateral TKA. The study by Benjamin et al comparing obese and nonobese patients undergoing unilateral and bilateral procedures is not equivalent to our study, as the bilateral procedures were 1-team, sequential procedures [22]. However, similar to our study, they were able to show a nonsignificant difference in wound and systemic complications between unilateral and bilateral obese groups, despite the approximate doubling of surgical time needed for sequential procedures as compared to unilateral procedures.

Wound problems have been among the most frequently cited complications of TKA in the obese population. Wilson et al were among the first to correlate obesity and wound infections in TKA [23]. Winiarsky et al found that TKA in obese patients, while commonly successful, is associated with increased rates of infection, wound complications, and medial collateral ligament avulsions [18]. In our series, there was a low overall rate of wound complications and infections, and there was no increase in this type of morbidity in patients undergoing the simultaneous bilateral procedure.

The use of blood transfusion is not without risk and is of concern to both the surgeon and patient [24]. A significant variation in reported blood transfusion rates for simultaneous and sequential bilateral total knee arthroplasty exists; rates reported have ranged from 17% to 91% [3,6,7,25-28]. This is likely due to differences in the criteria of reporting blood transfusion rates and blood loss, as well as the varying approaches used to manage acute blood loss. The rates of blood transfusion in our study decreased over time in both groups as policy was changed so that patients were treated symptomatically rather than automatically receiving a transfusion if hemoglobin levels dropped below particular levels (approximately 8 g/dL). Rates of autogenic preoperative donation also decreased over time in our study population, which may have lowered our transfusion rate over time. However, rates of transfusion in bilateral TKA patients in published studies have shown a universal increase in blood loss and transfusion rates, as would be expected with twice the surgical insult [3,6-8,11,25-28]. Lane et al observed that longer surgical duration in TKA is associated with higher crystalloid replacement, leading to a dilutional component of anemia [26]. Our bilateral group did have a significantly increased (*P *< .01) crystalloid replacement of approximately 10% over our unilateral group, which may also have contributed to a greater need for transfusion in patients undergoing the bilateral procedure. Our bilateral cohort did have a significantly higher (P < .01) transfusion rate without a significantly larger increase in postoperative hemoglobin levels (P = 0.23); this may be due to the increased crystalloid replacement, unseen postoperative blood loss, or some other unknown factor.

Greater emphasis is being placed on the cost benefit of various surgical procedures. Despite the fact that TKA has been shown to be an effective and cost-beneficial procedure, much attention continues to be paid to cost-cutting procedures [29-31]. The data contained in our study may have important consequences in this regard. The length of stay in the bilateral group was significantly longer than the unilateral group (3.72 vs. 3.30 days, *P *< .01), but staging the procedure for a bilateral situation would roughly double the unilateral time, causing significant increases in hospital inpatient stay costs. Operative time, which was also significantly increased in the bilateral group, would similarly be increased if a staged procedure would take place, leading to increased operating room expenses. However, 3 other important health system cost variables would not show a significant decrease in simultaneous 2-team TKA: the percentage of patients requiring transfusion, mean number of transfused units of packed red blood cells, and percentage of patients going to an extended-care facility at discharge. On the other hand, doubling the percentage of patients requiring extended-care facility treatment at discharge in the unilateral group to simulate staged procedures is only a very rough estimate, as level of deconditioning or decreased function as a result of a recent contralateral TKA is not taken into account, and may actually increase the use of extended-care facilities. This rough estimate prevents any conclusion regarding this statement in our study population. Reuben et al retrospectively compared the cost of unilateral vs. 1-team sequential bilateral TKA and noted a 36% cost reduction in the sequential bilateral total knee group as compared to a staged procdure [12]. Similarly, Brotherton et al determined that the overall hospital bill may be more than 50% greater when a staged TKA is performed rather than a sequential bilateral TKA [32].

Our study does have several inherent weaknesses. Its retrospective nature, as well as inability of randomization, could influence results by introducing bias. The small sample size of our groups could also introduce a type-II statistical error. However, because of the relatively low mortality of patients undergoing this procedure, an extremely larger and possibly impractical number of patients would have to be included to avoid such an error if evaluating mortality [33]. Although all of the patients were defined as obese by virtue of a BMI >30, there is likely a stratification of risk as patients reach more morbid levels of obesity, such as the patient with a BMI of 61.4 in our series. Additionally, although this series provides valuable outcome data, a comparison to non-obese patients at the same institution may generate beneficial data, as well. Another potential weakness is the fact that investigation into deep venous thrombosis was done only if the physicians had clinical suspicion in the perioperative period or as on an outpatient basis. Nonclinical deep venous thrombosis may have been missed and therefore the potential of bias from missing these nonsymptomatic thromboses is introduced.

## Conclusions

The effects of obesity on perioperative complications are of substantial concern to the orthopaedic surgeon. These concerns are heightened when performing a large and technically demanding procedure such as simultaneous bilateral TKA. The patients in this series experienced low rates of systemic complications and very few wound complications associated with their procedures. These results indicate that simultaneous, 2-team bilateral TKA in the obese patient can be a safe and successful procedure, with acceptably low rates of perioperative complications.

## Competing interests

The authors declare that they have no competing interests.

## Authors' contributions

STG, RNS designed the study. BCT, CD, JGM collected the data. BCT analyzed the data. BCT, CD, JGM prepared the manuscript. BCT, CD, JGM, STG, RNS ensured the accuracy of the data and analysis. All authors have read and approved the final manuscript.
